# *Lactobacillus reuteri* I5007 modulates tight junction protein expression in IPEC-J2 cells with LPS stimulation and in newborn piglets under normal conditions

**DOI:** 10.1186/s12866-015-0372-1

**Published:** 2015-02-15

**Authors:** Fengjuan Yang, Aina Wang, Xiangfang Zeng, Chengli Hou, Hong Liu, Shiyan Qiao

**Affiliations:** State Key Laboratory of Animal Nutrition, China Agricultural University, Beijing, 100193 China; Weifang Business Vocational College, Zhucheng, 262234 China

**Keywords:** *Lactobacillus*, Newborn piglets, Tight junction, IPEC-J2, Lipopolysaccharides

## Abstract

**Background:**

Tight junctions (TJs) maintain the intestinal mucosal barrier, dysfunction of which plays a vital role in the pathophysiology of a variety of gastrointestinal disorders. Previously, we have shown that *L. reuteri* I5007 maintained the gut epithelial barrier in newborn piglets. Here we aimed to decipher the influence of *L. reuteri* I5007 on tight junction (TJ) protein expression both *in vivo* and *in vitro*.

**Results:**

We found that *L. reuteri* I5007 significantly increased the protein abundance of intestinal epithelial claudin-1, occludin and zonula occluden-1 (ZO-1) in newborn piglets (orally administrated with 6 × 10^9^ CFU of *L. reuteri* I5007 daily for 14 days). *In vitro*, treatment with *L. reuteri* I5007 alone maintained the transepithelial electrical resistance (TEER) of IPEC-J2 cells with time. In addition, IPEC-J2 cells were stimulated with 1 μg/mL lipopolysaccharide (LPS) for 1, 4, 8, 12 or 24 h, following pre-treatment with *L. reuteri* I5007 or its culture supernatant for 2 h. The results showed that LPS time-dependently induced (significantly after 4 or 8 h) the expression of TNF-α and IL-6, and decreased TJ proteins, which was reversed by pre-treatment of *L. reuteri* I5007 or its culture supernatant.

**Conclusions:**

*L. reuteri* I5007 had beneficial effects on the expression of TJ proteins in newborn piglets and the *in-vitro* results showed this strain had a positive effect on TEER of cells and inhibited the reduction of TJ proteins expression induced by LPS. These findings indicated *L. reuteri* I5007 may have potential roles in protection TJ proteins in TJ-deficient conditions.

## Background

Diarrheal diseases in animals cause the livestock industry great economic loss and also represent a serious threat to farm animal welfare [1]. One of the main causes of diarrhea is gastrointestinal disorder and intestinal mucosa barrier dysfunction or “leaky gut” is reported to play a vital role in the pathophysiology of a variety of gastrointestinal disorders [2].

The intestinal mucosal barrier is maintained by tight junctions (TJs) [3], which are multiprotein complexes located around the apical end of the lateral membrane of the epithelial cells and seal the paracellular space between adjacent epithelial cells [4]. TJs function as a selective/semipermeable paracellular barrier, which regulates the transport of ions, water and solutes through the paracellular pathway [5,6]. The most important and critical components in the structural and functional organization of the TJs are occludin, zonula occluden-1 (ZO-1) and claudin-1 [7-9]. The importance of these TJ proteins has been demonstrated under many conditions. For example, increased intestinal permeability, which is thought to increase the load of bacterial and dietary antigens in the lamina propria, has been observed in early weaned pigs (21 days of age) apparently due to aberrant expression of the essential TJ proteins [10]. Hence, modulation of TJ function, particularly through increasing levels of occludin, ZO-1 and claudin-1, is a target for novel therapeutic or prophylactic treatments against a range of diseases.

Probiotics, defined as live microorganisms which, when consumed in adequate amounts as part of food, confer a health benefit on the host [11], are widely used in humans and animals. Since the European Union banned the use of antibiotics as growth promoters in 2006, the use of probiotic animal feed additives has increased as reported both *in vivo* [12,13] and *in vitro* [14,15]. Ingestion of probiotics has been shown to prevent or treat a variety of gut disorders [16,17], although the mechanisms through which they function are not completely known. However, a large amount of evidence suggests that one mechanism may be via protection or augmentation of the intestinal mucosal barrier function [17,18].

*Lactobacillus*, one of the most commonly used probiotic microorganisms [19,20], frequently occurs in the intestinal microflora of various vertebrates and exerts *Lactobacillus*-mediated barrier protection via modulation of TJs [21-23]. Previous studies have demonstrated that this genus significantly reduces the incidence and the severity of diarrhea [24] and helps maintain a functional mucosal barrier [25].

To test the hypothesis that natural *Lactobacillus* found in animal intestinal tract may be a potential source of probiotic bacteria for livestock. In 2003, our group isolated thousands of strains of *Lactobacillus* from the gastrointestinal tract GIT of eleven healthy weaned piglets from different pig farms. According to criteria including tolerance to heat, low pH, and bile salts, as well as storage stability and antagonism to pathogenic agents, four strains, *Lactobacillus fermentum* I5007, *L. gasseri* S1031, *L. reuteri* I2021 and *L. acidophilus* I021, were selected [26]. Subsequently, a series of *in vivo* and *in vitro* studies demonstrated that *L. fermentum* I5007 had the greatest adhesion [27], and showed beneficial effects on growth performance and antioxidative activity, as well as modulation of intestinal microbiota and mucin secretion in piglets [28-30]. Summarily, our work in the past decade indicates that *L. fermentum* I5007, now known as *L. reuteri* I5007 according whole-genome sequence of this strain [31], can be used as a potential probiotic. Recently, our group found that oral administration of *L. reuteri* I5007 favored intestinal development and maintained the gut epithelial barrier in neonatal piglets [28]. However, the mechanism through which it exerts functions on the gut epithelial barrier is not fully understood. The aim of the present study was to evaluate *L. reuteri* I5007-mediated intestinal mucosal barrier augmentation in a newborn piglet model and investigate the mechanism for modulation of TJs in a porcine jejunal epithelial cell line (IPEC-J2) challenged by lipopolysaccharides (LPS).

## Results

### Oral administration of *L. reuteri* I5007 enhanced the protein abundance of intestinal epithelial TJs in newborn piglets

**Figure 1:**
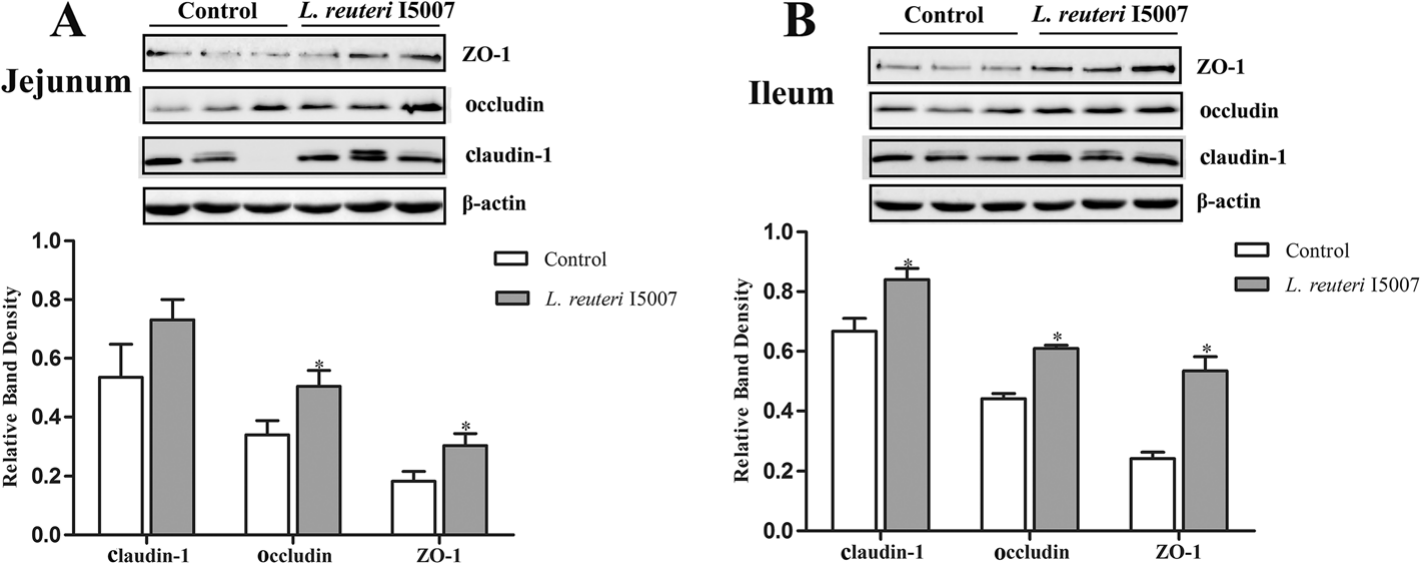
**Expression of TJ proteins in piglets following oral administration with** ***L. reuteri*** **I5007.** Four-day-old piglets were orally administrated with 6 × 10^9^ CFU of *L. reuteri* I5007 per day for 14 days. Subsequently, proteins were isolated from the intestinal tissues of piglets and then the expression of claudin-1, occludin and ZO-1 was measured using immunoblotting. **(A)** Protein levels of occludin and ZO-1 in jejunal tissue lysates were induced by *L. reuteri* I5007. **(B)** *L. reuteri* I5007 increased the expression of claudin-1, occludin and ZO-1 in the ileum. Data are represented as mean ± SEM, n = 6. *P < 0.05 compared with control piglets.

Twelve male newborn piglets (4 days old) were orally administrated with 0.1% sterile peptone solution (the control group) or 6 × 10^9^ CFU of *L. reuteri* I5007 (the *L. reuteri* I5007 group) daily for 14 days. The expression of TJ protein claudin-1, occludin and ZO-1 both in the jejunal and ileal epithelium were measured using immunoblotting to investigate the effect of *L. reuteri* I5007 on TJ function. Compared with control group, no significant difference was observed in the jejunal epithelial claudin-1 expression in the *L. reuteri* I5007 group, while oral administration of *L. reuteri* I5007 significantly increased the protein abundance of occludin and ZO-1 in jejunal epithelium of piglets (Figure 1A). However, all levels of these TJ proteins were distinctly higher in the ileum of piglets administrated with *L. reuteri* I5007 than those of control piglets (Figure 1B).

### *L. reuteri* I5007 maintained transepithelial electrical resistance (TEER) of IPEC-J2 cells

**Figure 2:**
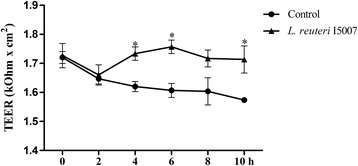
**Response of transepithelial electrical resistance (TEER) to** ***L. reuteri*** **I5007 in IPEC-J2 cells.** IPEC-J2 cells were treated with 3 × 10^7^ CFU/mL of *L. reuteri* I5007 cells alone for 0, 2, 4, 6, 8 and 10 h, and TEER values were measured, respectively. Treatment with *L. reuteri* I5007 induced a nice maintaince on TEER of IPEC-J2 cells with time. Results are represented as mean ± SEM. The experiment was repeated three times with quadruplicate wells in each assay. *P < 0.05 vs. control cells.

IPEC-J2 cell monolayers cultured for complete confluence were treated with *L. reuteri* I5007 cells (3 × 10^7^ CFU/mL) and TEER values were measured every two hours for 10 h (Figure 2). When compared with the untreated control, treatment with *L. reuteri* I5007 had no effect on TEER of IPEC-J2 cells during 0–2 h, but did lead to significantly higher values of TEER during 4–10 h. On the whole, an 8.67% decline of TEER values was caused during 10 h of the measurement, as seen for the control, while co-incubation with *L. reuteri* I5007 showed almost no decline (a 0.86% decline) in TEER values, indicating that *L. reuteri* I5007 had a positive maintaince on TEER of IPEC-J2 cells with time.

### *L. reuteri* I5007 differentially affected LPS-induced TNF-α and IL-6 mRNA expression in IPEC-J2 cells

To further elucidate the effects of *L. reuteri* I5007 on modulation of TJ function, the mRNA expression of inflammatory cytokines including TNF-α and IL-6 was determined in IPEC-J2 cells treated with LPS for 1, 4, 8, 12 or 24 h, following pre-treatment with *L. reuteri* I5007 for 2 h. According to a previous study of our group, *L. reuteri* I5007 could behave strong adhesion ability to monolayer cells by co-culture with cells for 1 h [27]. Resta-Lener and Barrett [32] showed enteroinvasive *Escherichia coli* evoked fall in TEER was prevented if cell monolayers were pretreated with live probiotics, and was also partially attenuated by exposure simultaneously to probiotics and enteroinvasive *E. coli*, but not if the cells were infected with enteroinvasive *E. coli* for one hour and then exposed to probiotics. Considering these circumstances, to investigate the protect effects of *L. reuteri* I5007 on TJ proteins, we appropriately extended the incubation time for pretreatment with *L. reuteri* I5007 to 2 h.

**Figure 3:**
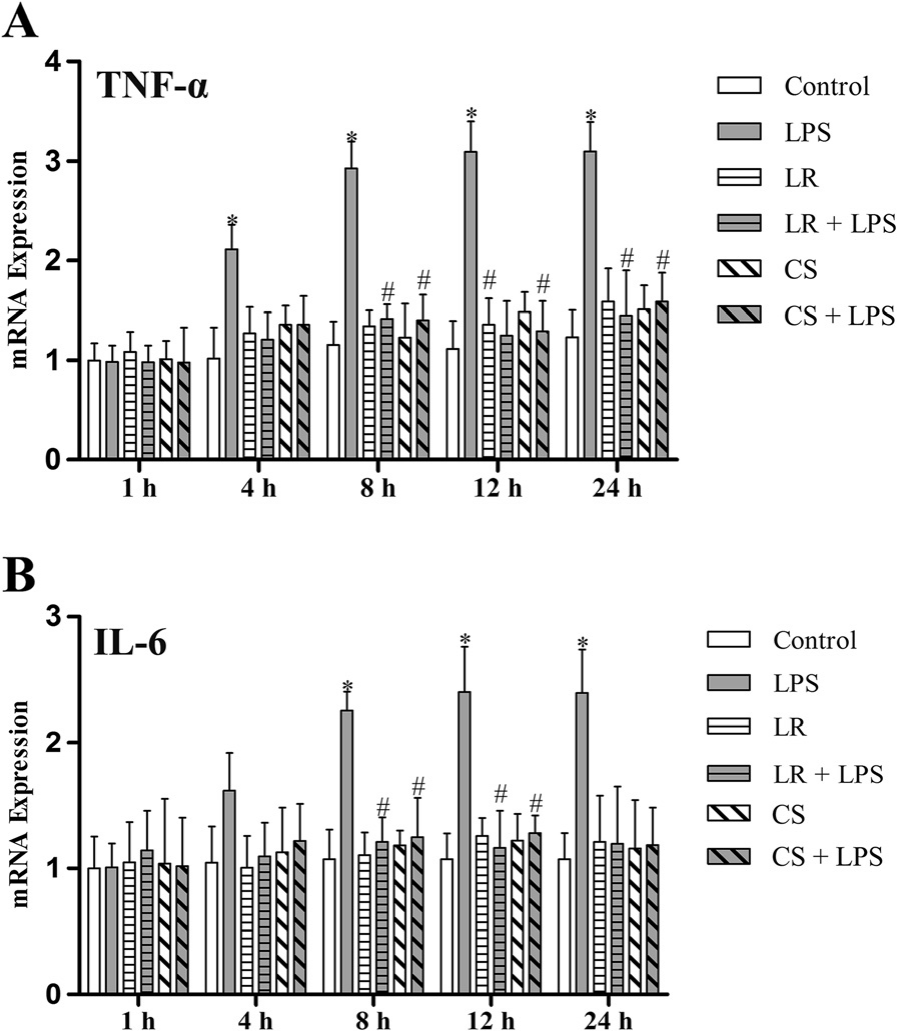
**mRNA expression of inflammatory cytokines in LPS-activated IPEC-J2 cells.** IPEC-J2 cells were pre-treated with either *L. reuteri* I5007 (LR) or *L. reuteri* I5007 culture supernatant (CS) 2 h prior to LPS-activation. Quantitative RT-PCR (qRT-PCR) analysis was performed to determine the mRNA expression of inflammatory cytokines after 1, 4. 8, 12 or 24 h treatment with LPS. **(A)** LPS affected no change in TNF-α expression at 1 h, but significantly increased TNF-α expression during 4–24 h. However, both *L. reuteri* I5007 and its culture supernatant had no effect on TNF-α expression at the different time points in the absence of LPS, while they inhibited or prevented LPS-induced expression of TNF-α. **(B)** There were obvious increases in IL-6 expression during 8–24 h, but not 1–4 h induced by LPS, while *L. reuteri* I5007 and its culture supernatant produced no change at any time compared with control cells. *L. reuteri* I5007 and its culture supernatant inhibited or prevented LPS-induced IL-6 expression. Results are represented as mean ± SEM, n = 3. *P < 0.05 vs. control cells, and #*P* < 0.05 vs. cells in the LPS group.

Figure 3A shows that LPS significantly increased TNF-α mRNA expression after 4 h, but not at 1 h. No significant differences were observed in the mRNA expression of TNF-α at 1 h among different treatments. However, TNF-α mRNA expression in LPS-stimulated cells was time-dependently increased compared with the control treatment, while after 12 h, the increase in TNF-α mRNA expression reached a plateau. Similarly, the mRNA expression of IL-6 (Figure 3B) was increased in IPEC-J2 cells treated with LPS after 8 h and was maintained until 12 h treatment. Collectively, treatment with *L. reuteri* I5007 in the absence of LPS had no effect on mRNA levels of TNF-α and IL-6 compared with the control group at different time points. However, in the presence of LPS, *L. reuteri* I5007 significantly decreased the mRNA expression of these two inflammatory cytokines, compared with the LPS group after 8 h.

### *L. reuteri* I5007 augmented LPS-induced TJ protein expression in IPEC-J2 cells

**Figure 4:**
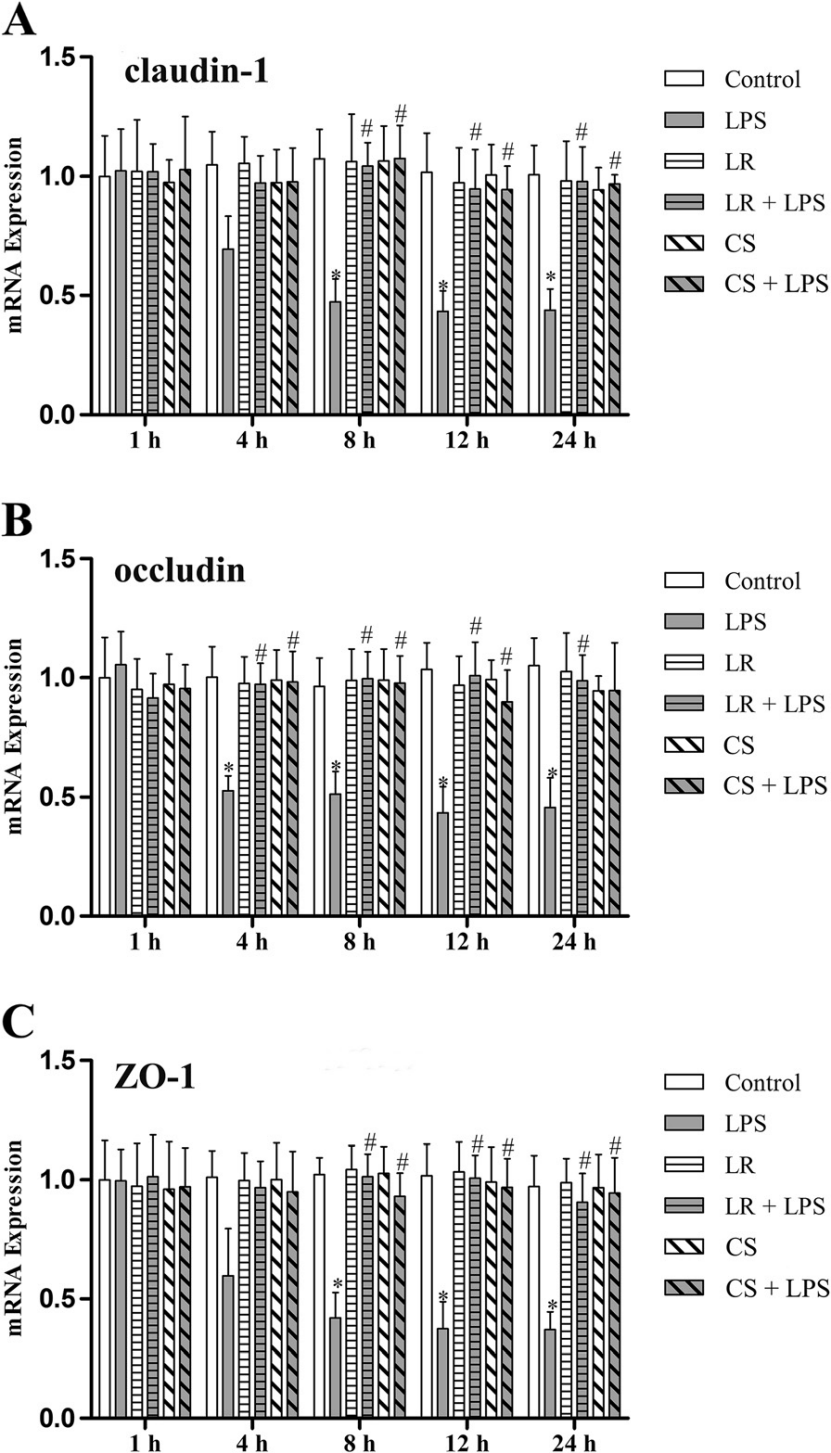
**mRNA expression of TJ proteins in LPS-activated IPEC-J2 cells. (A)** qRT-PCR analysis showed that LPS induced a decrease in mRNA expression of claudin-1 at 8, 12 and 24 h, while *L. reuteri* I5007 (LR) and *L. reuteri* I5007 culture supernatant (CS) effectively inhibited LPS-induced expression of claudin-1. **(B)** There was no change in occludin expression at 1 h, but a significant decrease after 4 h treatment with LPS. However, *L. reuteri* I5007 and its culture supernatant inhibited the decline. **(C)** LPS induced a distinct decrease in ZO-1 expression after 8 h, which was inhibited by *L. reuteri* I5007 and its culture supernatant. Results are represented as mean ± SEM, n = 3. *P < 0.05 vs. control cells, and #*P* < 0.05 vs. cells in the LPS group.

We further investigated the mRNA expression of claudin-1, occludin and ZO-1 in inflamed cultured intestinal cells. Treatment of IPEC-J2 cells with 1 μg/mL LPS had no effect on the mRNA expression of claudin-1 and ZO-1 during 1–4 h, but showed a significant decline during 8–24 h (Figure 4A and C). Similarly, compared with the control treatment, LPS time-dependently reduced occludin mRNA expression. In detail, no difference at 1 h, but decreases during 4–24 h (Figure 4B). However, there was no significant change in mRNA expression of all three TJ proteins in the other four groups versus the control group. Pre-treatment with *L. reuteri* I5007 significantly mitigated or prevented the reduction of mRNA expression of all three TJ proteins induced by LPS.

**Figure 5:**
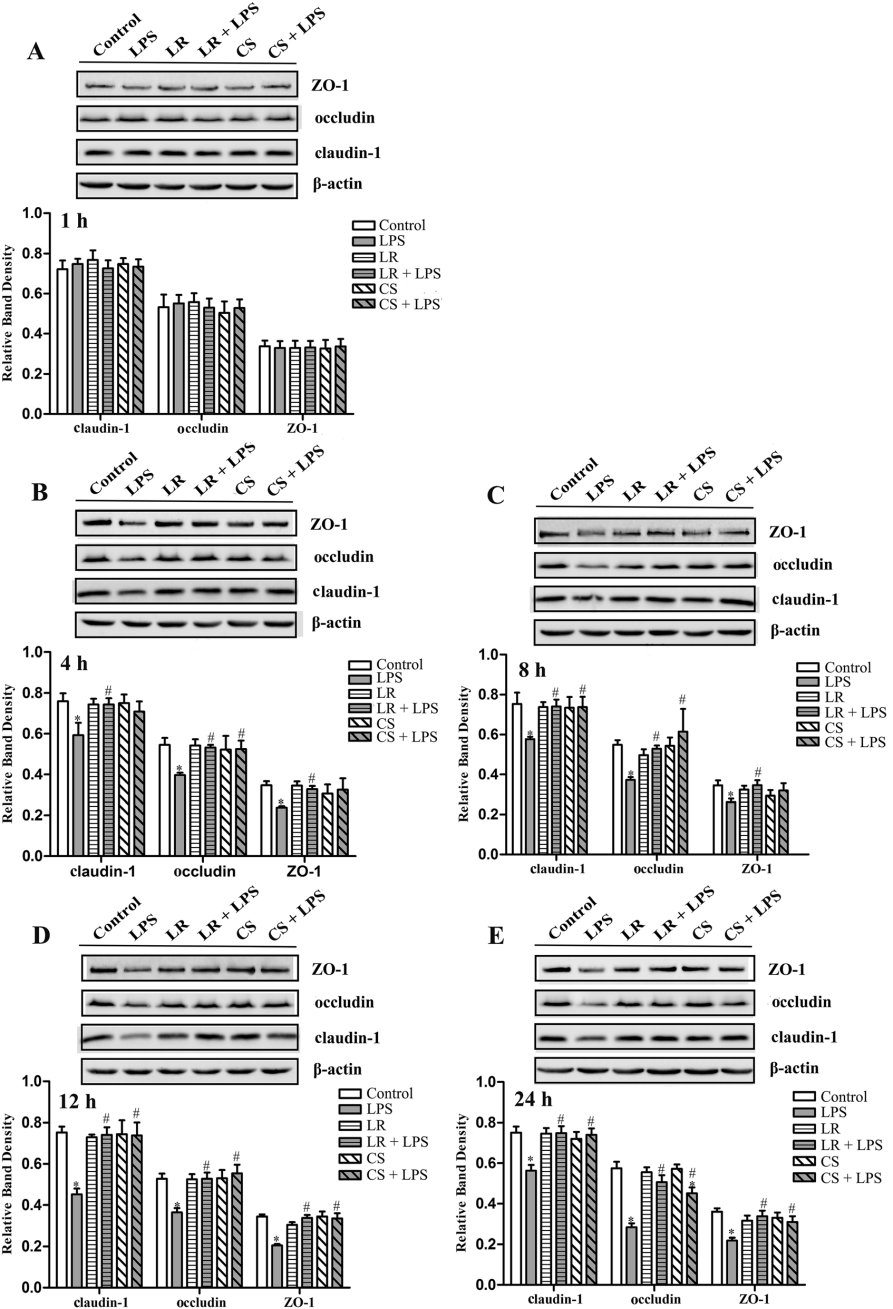
**Levels of TJ proteins in LPS-activated IPEC-J2 cells at different time points.** Proteins were isolated from IPEC-J2 cells and the expression of TJ proteins was assessed using immunoblotting. There was no significant difference in claudin-1, occludin and ZO-1 expression among the different treatments at 1 h **(A)**. LPS produced decreases in all three TJ proteins at 4 **(B)**, 8 **(C)**, 12 **(D)** and 24 **(E)** h. Although *L. reuteri* I5007 (LR) and its culture supernatant (CS) had no effect on the levels of TJ proteins in the absence of LPS, they inhibited LPS-induced levels of TJ proteins. Results are represented as mean ± SEM, n = 3. *P < 0.05 vs. control cells, and #*P* < 0.05 vs. cells in the LPS group.

We used immunoblotting to investigate the protein abundance of claudin-1, occludin and ZO-1. In agreement with the observation of mRNA expression of these TJ proteins, there was no significant difference in the levels of these TJ proteins in the other four groups (except for the LPS group) compared with the control group (Figure 5). However, LPS stimulation robustly decreased the levels of claudin-1, occludin and ZO-1 during 4–24 h (Figure 5B-E), but not at 1 h (Figure 5A). Treatment of IPEC-J2 cells using *L. reuteri* I5007 markedly induced higher levels of these proteins than those in cells treated with LPS during 4–24 h.

### *L. reuteri* I5007 culture supernatant differentially affected LPS-induced inflammatory cytokine and specific TJ protein expression in IPEC-J2 cells

To further elucidate whether the culture supernatant was involved in *L. reuteri* I5007-mediated improvement in TJ function, the culture supernatant of *L. reuteri* I5007 was also added to pre-treat IPEC-J2 cells. Compared with the control group, cells treated with *L. reuteri* I5007 culture supernatant in the absence of LPS caused no significant change in TNF-α and IL-6 mRNA expression. However, the culture supernatant of *L. reuteri* I5007 did reduce LPS-induced inflammatory cytokine expression during 8–24 h (Figure 3). In general, both mRNA expression and protein levels of claudin-1, occludin and ZO-1 in cells pre-treated with the culture supernatant *of L. reuteri* I5007 were higher than those in cells induced by LPS after 4 or 8 h (Figure 4 and Figure 5).

## Discussion

The defense response of the intestinal epithelium against pathogenic bacteria has been extensively explored, and evidence suggests that interactions between commensal bacteria and the host are involved in this process [33]. Thus, much attention has been paid to the effects of probiotics on gut barrier function. Some probiotics might improve or prevent gut barrier dysfunction caused by pathogenic bacteria or other toxic substances [22,23]. For example, *L. plantarum* significantly ameliorated phorbol ester-induced ZO-1 and occludin redistribution and increased permeability in Caco-2 cells [34]. Therefore, although the mechanisms of action of probiotics may vary, the vital mechanism of probiotics is regulating the expression of TJ proteins.

The effects of probiotics on gut barrier in animal models are commonly studied in rodents. For example, *L. helveticus* and *L. rhamnosus* prevented chronic stress induced intestinal abnormalities in rats, which received 7 days of these strains in the drinking water prior to either a water avoidance stress or a sham stress for one hour per day for ten consecutive days and remained on these probiotics during the duration of the study [35]. However, reports in pigs, especially in neonatal piglets, are rare. In the present study, newborn piglets were selected to study the effects of *L. reuteri* I5007 on the expression of TJ proteins in the intestinal epithelium. We showed that *L. reuteri* I5007 up-regulated the expression of jejunal epithelial occludin and ZO-1, and ileal epithelial claudin-1, occludin and ZO-1 in young piglets, indicating *L. reuteri* I5007 effectively improved the intestinal mucosal barrier function of newborn piglets. This was consistent with an earlier study [36] showing that *L. rhamnosus* accelerated maturation of the intestinal mucosal barrier in mice via up-regulating the protein level of claudin-1. Oral administration of VSL#3, a mixture of *Streptococcus thermophilus,* three strains of *Bifidobacterium* and four strains of *Lactobacillus*, in SAMP mice, obviously improved ileal epithelial permeability and increased the expression of occludin, but not claudin-1 or ZO-1 [21], which was not entirely consistent with our results. This might be due to different experimental conditions including strains of probiotic bacteria and animal models.

We next examined the effects of *L. reuteri* I5007 on the expression of inflammatory cytokines and specific TJ proteins in IPEC-J2 cells induced by LPS. LPS is an integral component of the gram-negative bacteria cell wall, capable of binding to Toll-like receptor-4 and activating a variety of protein kinase signaling pathways, subsequently generating inflammatory cytokines and other mediators [37]. Liu et al. [15] found that treatment of porcine intestinal epithelial cells (IPEC-J2) and rat intestinal epithelial cells (IEC-6) with LPS for 16 h markedly increased the secretion of IL-8, while co-culture with *L. reuteri* robustly inhibited LPS-induced overexpression of IL-8 in IPEC-J2 cells. In the present study, LPS induced increases in mRNA expression of TNF-α and IL-6 of IPEC-J2 cells after 4 or 8 h, and pre-treatment with *L. reuteri* I5007 significantly suppressed LPS-induced TNF-α and IL-6 expression. In addition, several studies have reported effects of probiotics on TNF-α and IL-6 expression induced by LPS, but the regulatory action differs in different species or even different strains. For instance, *L. reuteri* ACTT 6475 greatly inhibited the generation of TNF in LPS-activated human monocytoid THP1 cells, as an immunosuppressive action [38]. In contrast, *L. reuteri* ACTT 55730 stimulated TNF-α production, as an immunostimulatory action [39]. Moreover, Rachmilewitz et al. [40] observed that co-culture of mice macrophages (isolated from Balb/c mice) with DNA of VSL#3 bacteria significantly promoted IL-6 production.

TJs, apical-most component of the junctional complex in epithelial cells, determine the selective permeability along the paracellular pathway. claudin-1, occludin and ZO-1 are critical components of the TJs [41]. LPS challenge resulted in a distinct decrease in the levels of ZO-1 and ZO-2, and induced the redistribution of ZO-2 in human corneal epithelial cells [42]. Also, deterioration of the TJ proteins occludin and ZO-1 was observed in pulmonary cells of LPS-treated mice [43]. In agreement with previous studies, the present study found LPS reduced the levels of claudin-1, occludin and ZO-1 in IPEC-J2 cells. Our results also showed *L. reuteri* I5007 significantly attenuated the LPS-induced decline in claudin-1, occludin and ZO-1 expression. In fact, one of the effects probiotics exert is to promote the integrity of the intestinal mucosal barrier by affecting the expression and structure of TJ proteins. An earlier study, conducted in dextran sodium sulfate-induced colitis in mice, demonstrated the probiotic mixture VSL#3 prevented apoptosis and low expression of TJ proteins, and especially increased the ZO-1 level [44]. Khailova et al. [45] studied the effects of *Bifidobacterium Bifidum* in a rat model of necrotizing enterocolitis. The results showed that *B. Bifidum* improved intestinal integrity by normalizing the expression and localization of TJ and anherens junction proteins in the ileum compared to rats with neonatal necrotizing enterocolitis. Moreover, *Lactobacillus* and *Bifidobacterium* robustly suppressed *E. coli*-induced hyperpermeability and decreased ZO-1 expression *in vitro* [32].

A previous study showed that *Lactobacillus* and *Bifidobacterium* produced increases in the expression of the TJ proteins claudin-1 and occludin in normal human epidermal keratinocytes (NHEK) which had not been treated with any stressor [46]. However, it contrasts with our result that *L. reuteri* I5007 had no effect on the expression of specific TJ proteins in the absence of LPS. The *in-vitro* result seemingly showed differences from *in-vivo* finding that *L. reuteri* I5007 augmented TJ protein expression in piglets under normal conditions. It is well-known that GIT, in which microorganisms, toxins, digestive enzymes and other substances are all likely to cause damage, is the site of the largest and most complex environment in the mammalian host [47]. In this complex environment, piglets orally administrated with *L. reuteri* I5007 had higher expression of TJ proteins compared with control piglets which may be stimulated by complex factor in the GIT, indicating that *L. reuteri* I5007 exerted protective effects on TJs. *In vitro*, *L. reuteri* I5007 indeed prevented the LPS-induced decline in TJ protein expression, which further validated the protective effects of *L. reuteri* I5007 on TJ protein expression. Although *L. reuteri* I5007 behaved no effect in the absence of LPS, *in-vitro* results provide corroborative evidences that *L. reuteri* I5007 may play a positive role in protecting TJ proteins in dysfunctional or damaged GIT caused by pathogenic bacteria or other toxic substances [32,44]. In other words, *in-vivo* results together with *in-vitro* results imply that *L. reuteri* I5007 performs its protective effects by stabilization of synthesized TJ proteins or increasing new synthesis, and studies on a definite mechanism are needed.

TEER is a very important parameter of epithelial barrier function, which indicates variation of permeability and integrity of the cell monolayer [48,49]. In the present study, co-incubation with *L. reuteri* I5007 alone showed a nice maintaince on TEER of IPEC-J2 cells, consistent with an earlier study [46] showing that *L. rhamnosus GG* and *B. longum* increased TEER over control levels in NHEK cells. Furthermore, TEER in Caco-2 cells continued to decrease with time when treated with TNF-α, while increased in *L. plantarum* + TNF-α [50]. We also found the effects of LPS and *L. reuteri* I5007 on inflammatory cytokines and specific TJ protein expression were time dependent. There was no difference in the expression of TNF-α, IL-6 and TJ proteins among the different groups at 1–4 h. However, TNF-α and IL-6 mRNA expression in LPS-mediated cells increased and the levels of TJ proteins decreased with time compared with control cells after 4 or 8 h. Meanwhile, *L. reuteri* I5007 prevented the increased expression of TNF-α and IL-6 and decreased expression of TJ proteins induced by LPS. These findings were supported by work conducted by Sheth et al. [50], which evaluated the effect of LPS in normal rat cholangiocyte monolayers and showed LPS produced a time-dependent effect on TJ integrity and barrier function. Moreover, in LPS-activated Caco-2 cells model, TEER was minimized at 1 h after treatment with LPS and was sustained after that, while co-culture of *Lactobacillus* after activation with LPS for 3 h boosted TEER over time and was maximized at 24 h [51].

In the past, only live microorganisms were thought to exert probiotic effects, which may be why probiotics were defined as ‘live microorganisms which, when consumed in adequate amounts as part of food, confer a health benefit on the host’ [11]. As the effective ingredients of probiotics have not been fully revealed, more and more studies are further exploring this issue. Heat-killed preparations of the probiotic *L. rhamnosus* GG accelerated intestinal barrier maturation and induced claudin-3 expression [36]. Furthermore, treatment of NHEK with lysates of *Lactobacillus* and *Bifidobacterium* for 24 h produced significant increases in TEER and levels of TJ proteins in NHEK, suggesting lysates of microorganisms also can exert probiotic effects [46]. This study also showed peptidoglycan, a major ligand of gram-positive bacteria, induced increased TEER in keratinocytes, in agreement with a previous study [52], together raising the interesting possibility that the cell wall components from probiotic lysates may at least partially be responsible for the changes in TJ function. Herein, our results showed the culture supernatant of *L. reuteri* I5007 (metabolites or secreted bioactive factors from *L. reuteri* I5007) obviously attenuated or prevented high expression of inflammatory cytokines and low levels of TJ proteins induced by LPS, indicating the culture supernatant of *L. reuteri* I5007 played a role in *L. reuteri* I5007-mediated improvement in TJ function. Similarly, secreted bioactive factors from *B. infantis* significantly increased TEER and levels of occludin and ZO-1 in T84 cells [53]. Therefore, the culture supernatant of *L. reuteri* I5007 had a similar effect of *L. reuteri* I5007 on modulation of inflammatory cytokine and TJ protein expression, implying that substances secreted by *L. reuteri* I5007, including lactic acid, hydrogen peroxide, bacteriocin and exopolysaccharide, play a role in this effect. Whole genome sequencing result showed that genome of *L. reuteri* I5007 encoded two gene clusters for exopolysaccharide biosynthesis [31]. It may be proposed that secreted bioactive factors of *L. reuteri* I5007, especially exopolysaccharide interact with pattern recognition receptors of cells to induce activation of a series of signaling pathways [52] and subsequently regulate barrier function, including TJ function.

## Conclusions

Taken together, the data presented here suggest that *L. reuteri* I5007 could effectively increase the levels of specific TJ proteins in the intestinal epithelium, thereby promoting maturation of the intestinal mucosal barrier in formula-fed piglets. *L. reuteri* I5007 maintained TEER of IPEC-J2 cells and mitigated and even prevented LPS-induced inflammatory cytokine and TJ protein expression, and the effects might be derived from its culture supernatant and were time dependent. Since *L. reuteri* I5007 has a positive effect on TJ protein expression, it is possible that this stain plays a potential role in treatment or prevention of intestinal epithelial TJs deficiency in mammals or human beings.

## Methods

### Ethics statement

All procedures used in this experiment complied with the Animal Care Protocol which was approved by the China Agricultural University Animal Care and Use Committee (Beijing, China).

### Preparation of bacteria

*L. reuteri* I5007 was cultured to a stationary phase (about 24 h) in sterile Man Rogosa Sharpe (MRS) medium at 37°C in an anaerobic environment, then centrifuged at 5,000 × *g* for 10 min at 4°C. For animal administration, the centrifugal cells were resuspended in reconstituted skim milk (20% w/v) and immediately freeze-dried. Plate counts showed the freeze-dried powder contained 6 × 10^9^ colony forming units (CFU) of *L. reuteri* I5007 per gram. The bacteria powder was stored in sealed packets at a temperature of 4°C until used. During administration, 1 g of powder was dissolved in 3 mL 0.1% of sterile peptone solution for each piglet once a day. For cell culture assays, the culture supernatant of *L. reuteri* I5007 was reserved for subsequent treatment with a 10% (v/v) concentration. The centrifugal cells were washed three times in phosphate-buffered saline (PBS), and then resuspended in 1 mL PBS. Finally, 200 μL of bacterial resuspension solution was immediately used to treat cell cultures with a final concentration of 3 × 10^7^ CFU/mL according to a preliminary test (data not shown).

### Animals and experimental design

Twelve male Yorkshire × Landrace piglets which had been allowed to obtain colostrum from their dam for 48 h after birth were obtained from a commercial pig farm and transported to the Laboratory of Animal Metabolism at China Agricultural University (Beijing, China).

The piglets were individually housed in stainless steel cages (1.4 × 0.45 × 0.6 m^3^) in a temperature controlled nursery room (33 ± 1°C, 40%–60% relative humidity). On the third day of life, piglets were trained to suckle from pacifier bottles filled with milk replacer (Rosalac Instant, Bonilait Proteins, France). The milk replacer provided 45% lactose, 21.5% protein, 18.7% fat, 9.8% ash, 1.7% lysine, and sufficient vitamins and minerals to meet nutrient requirements. The milk replacer was dissolved in warm boiled water (w/v 1:5) to provide a similar dry matter concentration as sow’s milk. All the piglets were artificially fed every 4 h. On day four (equivalent to the first day of the trial), the piglets with an average body weight of 2.00 ± 0.31 kg were assigned into 2 groups with 6 piglets per treatment (a control group and a *L. reuteri* I5007 group) in a randomized complete block design according to their initial body weight. Each piglet in the *L. reuteri* I5007 group was orally administered with 6 × 10^9^ CFU *L. reuteri* I5007 dissolved in 3 mL 0.1% sterile peptone solution at a fixed time every day for 14 days, while the piglets in the control group were given the same volume of 0.1% sterile peptone solution.

On the morning of day 15, piglets were euthanized after an overnight fast and their abdominal cavities were opened to remove the gastrointestinal tract. The small intestine was carefully dissected from the mesentery and 5 cm segments of the jejunum and ileum were gently flushed with 0.9% physiological saline. Then the intestinal segments were frozen in liquid nitrogen and stored at -80°C for further analysis.

### Cell culture and treatment

IPEC-J2, a porcine intestinal epithelial cell line, was kindly provided by Dr. Bruce Schultz from Kansas State University (Manhattan, KS). IPEC-J2 cells were cultured in 6-well plates in DMEM/F12 medium (Thermo, Waltham, MA), supplemented with 5% fetal bovine serum (Gibco, Carlsbad, CA), 5 μg/L epidermal growth factor (ScienCell, Carlsbad, CA) and 0.1% 1X insulin-transferrin-sodium (ScienCell, Carlsbad, CA) at 37°C in a humidified 5% CO_2_ atmosphere. The culture was changed every other day.

TEER measurements were performed using a Millicell Electrical resistance system (Millipore, Billerica, MA). IPEC-J2 cells were seeded on the millicell membrance (12-wells, Millipore, Billerica, MA) cell inserts (Costar, Corning Inc., NY) at a density of 7 × 10^4^/cm^2^ and determined to be confluent at a TEER value of > 1 kOhm × cm^2^. When monolayer of cells was completely differentiated, cells were treated with 3 × 10^7^ CFU/mL of *L. reuteri* I5007 for 0, 2, 4, 6, 8 and 10 h and the TEER was measured respectively.

For measurements of inflammatory cytokine and TJ protein expression, IPEC-J2 cells were cultured in 6-well plates for complete confluence. Cells in each well were treated with 2 mL new medium containing 200 μL of *L. reuteri* I5007 resuspension solution (with a final concentration of 3 × 10^7^ CFU/mL) or its culture supernatant which had no bacteria in it (diluted 1:10 in basal medium) for 2 h. After rinsing in PBS three times, medium was then replaced with new medium containing 1 μg/mL LPS (*E. coli* 055:B5, Sigma, St. Louis, MO) based on the results of a preliminary test (data not shown). After 1, 4, 8, 12, or 24 h, cells were collected to extract total RNA and protein for follow-up studies. All experiments were repeated three times with duplicate wells within each individual run.

### RNA isolation and expression analysis by real-time PCR

Total RNA was isolated from the cells using a RNeasy kit (Qiagen, Hilden, Germany) according to the manufacturer’s instruction. RNA quality and quantity were determined by gel electrophoresis and a NanoDrop Spectrophotometer (P330, Implen, Germany). Subsequently, 1 μg RNA was reverse-transcribed to complementary DNA (cDNA) using a PrimeScript 1st Strand cDNA Synthesis Kit (Takala, Ostu, Japan) according to the manufacturer’s instructions. The gene-specific primer sequences are given in Table 1. Real-time PCR was performed using an Applied Biosystems 7500 Real-Time PCR System (Applied Biosystems, Singapore) with SYBR Green PCR Master Mix (Takala, Ostu, Japan), containing MgCl_2_, dNTP and Hotstar Taq polymerase. Briefly, copy numbers were calculated relative to a dilution series (1:10^7^ to 1:1) of the respective reference plasmids which contained the cloned RT-PCR products obtained with these primers. The PCR system consisted of 5.0 μL of YBR Green qPCR Mix, 1.0 μL of cDNA, 0.25 μmol of each primer, and 3.6 μL of double distilled water in a final volume of 20 μL. Each sample was determined in triplicate and the housekeeping gene, β-actin, was used as the internal standard for the PCR reaction.

**Table 1:** **Primers used for real-time PCR**

**Genes**	**Primers**	**Sequences (5'-3')**	**Size (bp)**	**Tm (°C)**
TNF-α	Forward	ATTCAGGGATGTGTGGCCTG	120	62
	Reverse	CCAGATGTCCCAGGTTGCAT		
IL-6	Forward	TGGATAAGCTGCAGTCACAG	109	54
	Reverse	ATTATCCGAATGGCCCTCAG		
Claudin-1	Forward	GCAGCAGCTTCTTGCTTCTC	664	58
	Reverse	CTGGCATTGACTGGGGTCAT		
Occludin	Forward	ATCAACAAAGGCAACTCT	157	50
	Reverse	GCAGCAGCCATGTACTCT		
ZO-1	Forward	GAGTTTGATAGTGGCGTT	298	50
	Reverse	GTGGGAGGATGCTGTTGT		
β-actin	Forward	TGCGGGACATCAAGGAGAAG	216	60
	Reverse	AGTTGAAGGTGGTCTCGTGG		

### Extraction of protein and immunoblotting

Total protein was extracted from the intestinal tissues or IPEC-J2 cells using lysis buffer (150 mM NaCl, 1% Triton X-100, 0.5% sodium deoxycholate, 0.1% SDS, 50 mM Tris-HCl at pH 7.4, plus a protease inhibitor cocktail purchased from Applygene, Beijing, China). Briefly, 0.02 g of each frozen jejunum and ileum sample were powdered under liquid nitrogen, and lysed in lysis buffer containing protease inhibitors. IPEC-J2 cells in each well were scraped into 100 μL of ice-cold lysis buffer containing protease inhibitors and then incubated on ice for 30 min. The lysed samples were centrifuged at 10,000 × *g* for 5 min at 4°C and the supernatant was collected. Total protein concentrations were determined using a BCA Protein Assay Kit (Pierce, Rockford, IL). Equal amounts of proteins (30 μg) were electrophoresed on SDS polyacrylamide gel, and proteins were electrophoretically transferred onto polyvinylidene difluoride (PVDF) membranes (Millipore, Bedford, MA). These were then blocked in 5% skim milk, and incubated (overnight at 4°C) with primary antibodies against claudin-1 (Sigma, St. Louis, MO), occludin (Abcam, Cambridge, United Kingdom), ZO-1 (Santa Cruz Biotechnology, Santa Cruz, CA), and β-actin (Cell Signaling Technology, Danvers, MA). The membranes were subsequently washed and incubated (1 h at room temperature) with horseradish peroxidase-conjugated secondary antibodies (Zhongsan Gold Bridge, Beijing, China). The immunoblots were developed with the Western blot Luminence Reagent (Santa Cruz Biotechnology, Santa Cruz, CA), and exposed by AlphaImager 2200 (Alpha Innotech, San Leandro, CA) automatically. Band densities were quantified using AlphaImager 2200 (Alpha Innotech, San Leandro, CA).

### Statistical analysis

All data were statistically analyzed using SPSS Software Version 17. The statistical significance of differences of *in-vivo* data was determined by Student’s *t*-test. Each piglet served as an experimental unit. One way analysis of variance (ANOVA) was used with Student-Neuman-Keuls (SNK) as a multiple comparsion test to analyse *in-vitro* data, except for data of TEER, which was analyzed by Student’s *t*-test. A *P* value < 0.05 was considered significant. Results are expressed as mean ± standard error of the mean (SEM).

## Abbreviations

TJ: Tight junction
ZO-1: Zonula occluden-1
LPS: Lipopolysaccharide
CFU: Colony forming units
NHEK: Normal human epidermal keratinocytes
TEER: Transepithelial electrical resistance
